# Evaluation of Spectral X-Ray Imaging for Panoramic Dental Images Based on a Simulation Framework

**DOI:** 10.1007/s10278-023-00940-8

**Published:** 2024-01-12

**Authors:** Daniel Berthe, Anna Kolb, Abdulrahman Rabi, Thorsten Sellerer, Villseveri Somerkivi, Georg Constantin Feuerriegel, Andreas Philipp Sauter, Felix Meurer, York Hämisch, Tuomas Pantsar, Henrik Lohman, Daniela Pfeiffer, Franz Pfeiffer

**Affiliations:** 1 Department of Physics, School of Natural Sciences, Technical University of Munich, 85748 Garching, Germany; 2 Munich Institute of Biomedical Engineering, Technical University of Munich, Boltzmannstr. 11, 85748 Garching, Germany; 3 Department of Diagnostic and Interventional Radiology, School of Medicine, Klinikum rechts der Isar, Technical University of Munich, 81675 Munich, Germany; 4 Planmeca Oy, 00880 Helsinki, Finland; 5 Varex Imaging Corp, Salt Lake City, 84104 USA; 6 Institute for Advanced Study, Technical University of Munich, 85748 Garching, Germany

**Keywords:** Panoramic radiography, Dental digital radiography, Spectral imaging, Photon counting detectors, Material decomposition

## Abstract

Modern photon counting detectors allow the calculation of virtual monoenergetic or material decomposed X-ray images but are not yet used for dental panoramic radiography systems. To assess the diagnostic potential and image quality of photon counting detectors in dental panoramic radiography, ethics approval from the local ethics committee was obtained for this retrospective study. Conventional CT scans of the head and neck region were segmented into bone and soft tissue. The resulting datasets were used to calculate panoramic equivalent thickness bone and soft tissue images by forward projection, using a geometry like that of conventional panoramic radiographic systems. The panoramic equivalent thickness images were utilized to generate synthetic conventional panoramic radiographs and panoramic virtual monoenergetic radiographs at various energies. The conventional, two virtual monoenergetic images at 40 keV and 60 keV, and material-separated bone and soft tissue panoramic equivalent thickness X-ray images simulated from 17 head CTs were evaluated in a reader study involving three experienced radiologists regarding their diagnostic value and image quality. Compared to conventional panoramic radiographs, the material-separated bone panoramic equivalent thickness image exhibits a higher image quality and diagnostic value in assessing the bone structure $\left(p<.001\right)$ and details such as teeth or root canals $\left(p<.001\right)$. Panoramic virtual monoenergetic radiographs do not show a significant advantage over conventional panoramic radiographs. The conducted reader study shows the potential of spectral X-ray imaging for dental panoramic imaging to improve the diagnostic value and image quality.

## Introduction

Photon counting detectors are emerging as a promising technology in medical imaging, offering several advantages such as improved contrast and resolution, no electronic noise, and reduced radiation dose [1]. Additionally, the detectors can sort detected photons into several energy bins, enabling the calculation of virtual monoenergetic or material-decomposed images [2]. However, while these detectors have already found clinical application in some areas of radiology [3], their potential benefits for dental imaging still need to be thoroughly evaluated. Dental panoramic radiography is a widely used diagnostic tool [4] to assess the maxilla, the mandible, and the teeth and any lesions in these structures [5]. However, its image quality and diagnostic value can be limited by artefacts resulting from factors such as improper tongue placement or dental implants [6]. In this context, photon counting detectors could provide significant advantages by allowing the calculation of virtual monoenergetic or material-decomposed panoramic images [7].

Monoenergetic images have proven beneficial in CT, by reducing beam hardening and enhancing the contrast after a measurement tailored to specific subjects by changing the virtual energy [8].

Furthermore, the material-separated bone image can eliminate superimposed soft tissue structures like the tongue, lingual tonsils, or the soft palate [9], often making assessing bony structures difficult. On the other hand, the soft tissue image can potentially be used to evaluate soft tissue-related diseases, like maxillary sinusitis, that conventional panoramic images mostly cannot resolve [10]. Thus, the question arises whether not only CT, which is mostly used for oral and maxillofacial surgery in the dental context, but also panoramic dental radiography could benefit from the additional spectral information. However, before photon counting detectors can be applied in clinical practice, their diagnostic and image quality potential must be evaluated.

In this study, we present a reader study of simulated panoramic images to evaluate the potential benefits of photon counting detectors in dental panoramic radiography. We assess the diagnostic value and image quality of panoramic virtual monoenergetic images (PVMIs) at 40 keV and 60 keV, as well as material-separated bone and soft tissue panoramic equivalent thickness images (PETIs), compared to synthetic conventional panoramic radiographs. In a previous study [13], the used simulation framework, which simulates the different spectral panoramic images from conventional non-spectral CT data, was published.

## Materials and Methods

### Simulation Framework

**Fig. 1:**
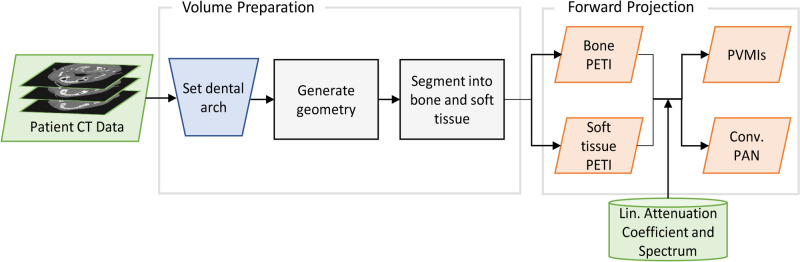
Workflow for generating panoramic images from patient CT data. The CT data is used to generate the source and detector movement for the forward projection of the segmented data. This yields the panoramic equivalent bone and soft tissue image. From this, the synthetic conventional and virtual monoenergetic images are derived with the linear attenuation coefficients and source spectrum

The simulation framework consists of three main steps which are visualized in Fig. 1. The framework requires conventional non-spectral head CT data. Ideally, photon-counting CT data would be preferred, but the lack of sufficient patient or phantom data would make conducting a meaningful statistical analysis impossible. First, the head CT data is used to define the dental arch; from this, the simulated photon counting detector and source movement are calculated. Secondly, a bone enhancement filter [11] is applied to the CT data, which is then segmented into bone, soft tissue, and air by thresholding. Next, the bone and soft tissue segmentations are forward projected with the simulated detector and source movement to obtain the panoramic equivalent thickness images (PETI) for bone and soft tissue. Each pixel value of the PETIs represents the projected thickness of the respective material. Finally, the synthetic conventional panoramic image (PAN) is generated by obtaining the linear attenuation coefficients of both basis materials from [12] and simulating a tungsten X-ray source spectrum. Then, the Beer-Lambert law is used to calculate the expected photon counts for each pixel. The panoramic virtual monoenergetic images (PVMIs) are generated by multiplying the PETIs with their corresponding linear attenuation coefficients and adding them together. The PVMI shows a synthetic representation of the image as if it was taken with a monochromatic X-ray source. The virtual X-ray energy can be adjusted after the measurement to tailor it, e.g. to specific tissue types. The resulting images were not further post-processed. For a more comprehensive understanding of the simulation framework and a detailed comparison with experimentally obtained images which validate the simulation, please refer to [13].

### Reader Study

The image evaluation was performed by three radiologists with experience of 8, 8 and 6 years in musculoskeletal radiology and 3, 4 and 6 years training in head and neck imaging. The evaluation focussed on the question whether virtual monoenergetic images and decomposed images into material components show a diagnostic benefit in dental panoramic radiography. A total of 17 different patient CT datasets were used to simulate the synthetic conventional PAN, PETI and PVMI for each patient. All images were rated in reading rooms with lighting below 50 Lux according to the standards for radiological reporting and on medical displays (EIZO Color medical LCD Monitor Modell RX250 and Modell RX350) calibrated to the DICOM GSDF with regular constancy tests. The radiologists evaluated the PANs, PVMIs and bone PETIs individually, while the soft tissue PETIs were assessed in combination with the respective bone PETIs side by side. The images were rated on a scale of one to five in the categories listed in Table 1. Since the study focuses on assessing bone structures, mainly teeth and root canals were considered as diagnostic details. All images were pre-windowed, but the radiologists could adapt the windowing, zoom and pan during the evaluation.

**Table 1:** Evaluation criteria and rating scale for the reader study

**Image quality for evaluation of bone structures**				
5 = excellent	4 = good	3 = moderate	2 = bad	1 = not appropriate/applicable
**Visualization of diagnostic details such as teeth or root canals**				
5 = excellent	4 = good	3 = moderate	2 = bad	1 = not appropriate/applicable
**Artefacts**				
5 = no	4 = minor	3 = major	2 = bad	1 = unacceptable
**Overall image quality**				
5 = excellent	4 = good	3 = moderate	2 = bad	1 = unacceptable
**Diagnostic acceptability**				
5 = fully acceptable	4 = probably acceptable	3 = acceptable only under limited conditions	2 = bad	1 = unacceptable

All images were presented to the readers randomly to keep the ratings independent of the previous rating session. Reader 1 rated all image categories within one day, whereas reader 2 and reader 3 assessed the images within several days or weeks.

### Statistical Analysis

We performed a Wilcoxon signed-rank test using IBM SPSS (version 29.0.0.0) to compare the ratings of the PETIs and PVMIs to those of the synthetic conventional PAN images. This allowed us to determine whether there was a statistically significant difference at the 0.05 level of significance. The Wilcoxon signed-rank test requirements are satisfied due to the ordinal nature of the scores, their non-normal distribution, and their interdependence. The dependence of the data is because the three image modalities are derived from the same CT data sets.

## Results

### Patient Data

Ethics approval from the local ethics committee (Ethics Commission of Technical University of Munich, Germany) was obtained for this retrospective study. The datasets were taken with a Philips iQon CT, and overall, 17 head CTs were included in the study. For all scans, the same facial skull protocol was used with an acceleration voltage of 120 kVp and a slice thickness of 0.67 mm. To avoid reconstruction artefacts, only patient data without crowns or implants and low noise in the CT data were selected. Furthermore, attention was paid that the cervical spine was stretched to avoid a spine shadow. Normally, before a PAN scan, it is ensured that the tongue is pressed against the palate and the teeth are displayed without overlapping.  Due to the limited number of patient data, this was neglected. Apart from this, no anatomical or disease condition was defined as an exclusion or inclusion criterion.

### Patient Images

**Fig. 2:**
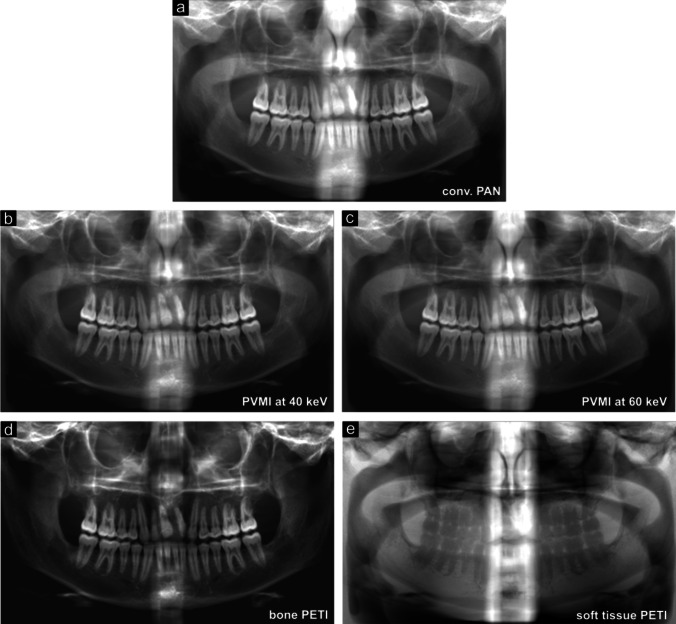
Exemplary simulated panoramic images. For each patient a conventional panoramic image (**a**), both panoramic virtual monoenergetic images at 40 keV (**b**) and 60 keV (**c**) and the material-separated bone (**d**) and soft tissue (**e**) panoramic image are simulated. The here depicted images were generated from the same CT dataset

Figure 2 shows examples of the simulated PAN image (a), PVMIs at 40 keV (b) and 60 keV (c), and the material-separated bone (d) and soft tissue (e) PETI of one patient. The shown images are pre-windowed as the radiologists originally received them. We visually adjusted the windowing of each image to achieve maximum contrast, but the radiologists could adjust it themselves as desired.

### Reader Scoring

**Fig. 3:**
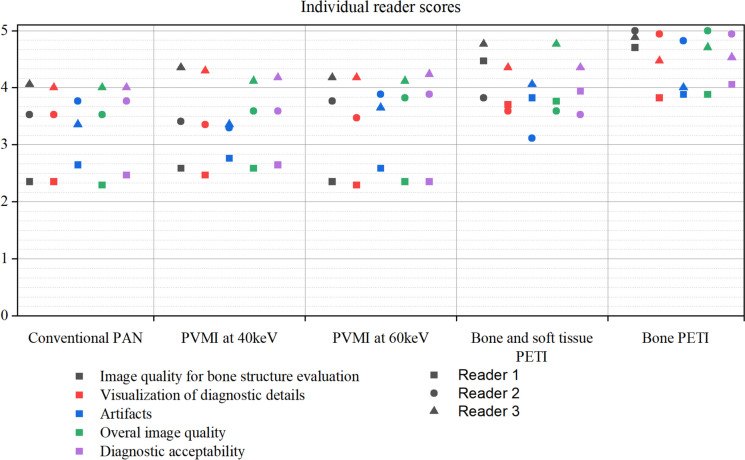
Individual scores of the reader in the study. The individual ratings of the readers are given for each image category concerning the different qualitative criteria

The resulting scores of the three readers are listed in Table 2 and visualized with a scatter plot in Fig. 3.

**Table 2:** Reader study results. The table lists the results of the readers for all image types and criteria, as well as the average score and the *p*-value regarding the conventional panoramic radiograph (PAN). The bone panoramic equivalent image (PETI) was rated significantly highest in all categories. Both panoramic virtual monoenergetic images (PVMI) at 40 keV and 60 keV are not rated significantly better than the conventional panoramic images

	**Reader 1**	**Reader 2**	**Reader 3**	**Average score**	**Significance**
**Conventional pan (50 points)**					
Image quality for evaluation of bone structures	2.35 ± 0.49	3.53 ± 0.51	4.06 ± 0.56	3.31 ± 0.88	-
Visualization of diagnostic details	2.35 ± 0.49	3.53 ± 0.51	4.00 ± 0.50	3.29 ± 0.86	-
Artefacts	2.65 ± 0.49	3.76 ± 0.44	3.35 ± 0.61	3.25 ± 0.69	-
Overall image quality	2.29 ± 0.47	3.53 ± 0.51	4.00 ± 0.50	3.27 ± 0.87	-
Diagnostic acceptability	2.47 ± 0.51	3.76 ± 0.44	4.00 ± 0.50	3.41 ± 0.83	-
**PVMI at 40 keV (51 points)**					
Image quality for evaluation of bone structures	2.59 ± 0.51	3.41 ± 0.62	4.35 ± 0.49	3.45 ± 0.90	.12
Visualization of diagnostic details	2.47 ± 0.51	3.35 ± 0.61	4.29 ± 0.47	3.37 ± 0.92	.43
Artefacts	2.76 ± 0.44	3.29 ± 0.59	3.35 ± 0.49	3.14 ± 0.57	.24
Overall image quality	2.59 ± 0.51	3.59 ± 0.51	4.12 ± 0.33	3.43 ± 0.78	.03
Diagnostic acceptability	2.65 ± 0.49	3.59 ± 0.51	4.18 ± 0.39	3.47 ± 0.78	.44
**PVMI at 60 keV (51 points)**					
Image quality for evaluation of bone structures	2.35 ± 0.49	3.76 ± 0.44	4.18 ± 0.53	3.43 ± 0.92	.20
Visualization of diagnostic details	2.29 ± 0.59	3.47 ± 0.51	4.18 ± 0.53	3.31 ± 0.95	.84
Artefacts	2.59 ± 0.51	3.88 ± 0.33	3.65 ± 0.49	3.37 ± 0.72	.16
Overall image quality	2.35 ± 0.49	3.82 ± 0.39	4.12 ± 0.33	3.43 ± 0.88	.74
Diagnostic acceptability	2.35 ± 0.49	3.88 ± 0.33	4.24 ± 0.44	3.49 ± 0.92	.32
**Bone PETI (68 points)**					
Image quality for evaluation of bone structures	4.71 ± 0.47	5.00 ± 0.00	4.88 ± 0.33	4.86 ± 0.35	< .001
Visualization of diagnostic details	3.82 ± 0.64	4.94 ± 0.24	4.47 ± 0.51	4.41 ± 0.67	< .001
Artefacts	3.88 ± 0.33	4.82 ± 0.39	4.00 ± 0.00	4.24 ± 0.51	< .001
Overall image quality	3.88 ± 0.60	5.00 ± 0.00	4.71 ± 0.47	4.53 ± 0.64	< .001
Diagnostic acceptability	4.06 ± 0.43	4.94 ± 0.24	4.53 ± 0.51	4.51 ± 0.54	< .001
**Bone and soft tissue PETI (60 points)**					
Image quality for evaluation of bone structures	4.47 ± 0.62	3.82 ± 0.39	4.76 ± 0.44	4.35 ± 0.63	< .001
Visualization of diagnostic details	3.71 ± 0.59	3.59 ± 0.51	4.35 ± 0.49	3.88 ± 0.62	< .001
Artefacts	3.82 ± 0.39	3.12 ± 0.49	4.06 ± 0.24	3.67 ± 0.55	< .001
Overall image quality	3.76 ± 0.56	3.59 ± 0.51	4.76 ± 0.44	4.04 ± 0.72	< .001
Diagnostic acceptability	3.94 ± 0.43	3.53 ± 0.51	4.35 ± 0.49	3.94 ± 0.58	< .001

In general, the PVMIs were rated similarly and only slightly higher than the synthetic conventional PAN. However, the difference is only marginal and insignificant as the readers differ in their assessment.

The Bone PETI obtained the highest rating (*p* < 0.001) and the lowest standard deviation in all rating categories. It was rated to have the most diagnostic value to assess details and evaluate bone structures. Also, according to the readers, the bone PETI showed the highest image quality with the lowest artefacts. Compared to the conventional PAN and both PETIs, the standard deviation of the bone PETI is the lowest, which highlights the unity of the readers.

The combination of the bone and soft tissue PETI side by side received slightly lower ratings than the bone PETI. Especially, the artefacts appear more prominent for the combined images. However, the image combination still has a significantly higher image quality and diagnostic assessment than the synthetic conventional PANs and the PVMIs.

By comparing the sum of each rating category for all image modalities, the bone PETI is overall rated highest with 67.65 out of 75 points, followed by the combination of the bone PETI next to the soft tissue PETI with 59.65 points. The PVMIs at 40 keV and 60 keV received 50.59 and 51.12 points, respectively, similar to the conventional PAN, with 49.65 out of 75 points.

## Discussion

The reader study results indicate that photon counting detectors are a promising technique for dental panoramic imaging.

The material decomposition effectively removes superimposed tissue structures from the bone image, consequently improving overall image quality and the diagnostic value of the PETI. Furthermore, the proposed method can mitigate positioning artefacts due to improper tongue placement during panoramic radiography. These artefacts, which appear as dark shadows caused by air pockets trapped between the tongue and palate, are the most common errors encountered in panoramic imaging [14].

In this study, the evaluation of spectral imaging for dental panoramic radiography was focused on assessing bone structures, teeth, and roots. Therefore, the additional information provided by the soft tissue image did not benefit the purpose of the study. Nevertheless, insights from photon counting CT scans have demonstrated the significant diagnostic advantages of material separation for soft tissue-related diseases [15]. Therefore, it is worth noting that soft tissue-related diseases such as sinusitis or pulpitis are of potential interest for spectral imaging studies. However, obtaining an appropriate sample size of patients for such studies may present a challenge.

Although the PVMIs generated at 40 keV and 60 keV did not offer significant advantages over synthetic conventional PANs, it is worth noting that the energy values of the PVMIs were selected based on the authors' subjective visual assessment. While it is possible that other energy values could lead to improved results, it is unlikely that they would surpass the significant benefits of the bone PETI.

The simulation considers an ideal detector response and did not incorporate scattering or noise, as the focus was on evaluating whether spectral imaging can improve image quality at all, regardless of the technical implementation. Further experimental validation of the results of this reader study is therefore required to confirm the promising potential of photon counting detectors for dental panoramic imaging. In addition, only CT datasets without artefacts and with low noise were used to obtain the best possible panoramic images to facilitate scoring by the readers. However, this does not influence the evaluation results, since only the relative scoring between the image modalities is of interest and not the overall image quality of the simulation framework.

Another benefit of photon counting detectors, which is not covered in the work, is the drastic resolution and sharpness increase compared to flat panel detectors [16]. Here, the resolution of the simulated images is limited by the voxel size of the CT data and is consequently far below of what modern photon counting detectors can resolve. However, the resolution is the same for all modalities, enabling a fair comparison of the images. The first results from commercially available photon counting CT systems proof its benefit for medical imaging. The photon counting detectors provide a higher resolution, better dose efficiency [16], and enable material decomposition. With our simulation, we could show that photon counting detectors can bring a significant benefit for dental imaging as well, especially in terms of image quality. This in turn enables a more precise diagnosis and a further reduction in the radiation dose to the patient with the same image quality. Another application of the spectral information can be to identify dental restorative dental materials [17] or to assess the bone mineral density of patients [18].

## Conclusion

In summary, photon counting detectors can significantly enhance the diagnostic value of dental panoramic imaging by effectively removing superimposed tissue structures and reducing positioning artefacts. This approach could consequently enhance the diagnostic value of panoramic radiography significantly, but further experimental validation is required.

## Data Availability

The data that support the findings of this study are available from the corresponding author, upon reasonable request.

## Abbreviations

PAN: Panoramic radiograph
PETI: Panoramic equivalent thickness image
PVMI: Panoramic virtual monoenergetic image
